# Supplementary material for: Learning curve and surgical outcome of robotic assisted colorectal surgery with ERAS program
Source: Sci Rep. 2022 Nov 29;12:20566. doi: 10.1038/s41598-022-24665-w (PMC9709162; doi:10.1038/s41598-022-24665-w)
Supplement: Supplementary file 2 — Supplementary Information 2. [file 41598_2022_24665_MOESM2_ESM.zip › RAL Raw data-Ñ[▒K/091/HS_ñw╝╨░O▒Kñσ.pdf]

正式摘要(電子簽章：000000218842040)

W95 / W95 ☐ 英文標題(注意:無英文姓名-請補病人基本檔!) ☐【讀取】憑證

臺中榮民總醫院

出院病歷摘要

轉入號 : 轉出號 :

[redacted]

**(8)**流水編號	
**(9)**入院日期	110/06/09 (2021) 直腸外科 W95 - 10
**(10)**轉科日期	
**(11)**出院日期	110/06/15 (2021) 住院天數計 6 日

診斷

- 入院 :Rectal CA, cT3N1M0, stage IIIb, s/p biopsy on 2021/04/29, adenocarcinoma, MD, s/p neoadjuvant CCRT
- 出院 :Rectal CA, cT3N1M0, stage IIIb, s/p biopsy on 2021/04/29, adenocarcinoma, MD, s/p neoadjuvant CCRT, s/p robotic assisted low anterior resection + vaginal wall tumor excision on 2021/06/10

主訴

- Rectal CA

病史

- This 50 y/o female with a history of

  Rectal CA, cT3N1M0, stage IIIb, s/p biopsy on 2021/04/29, adenocarcinoma, MD

  went to CRS OPD for help where neoadjuvant CCRT was suggested. After neoadjuvant CCRT, operation was suggested. As a result, she was admitted for op.

體檢發現

-
  - General appearance :
    - easy looking , conscious clear , without cardiopulmonary distress
    - Vital signs自動帶入date 體溫(BT) 脈搏(P) 呼吸(R) 血壓(BP) 身高(BH) 體重(BW) BMI
      20210609 35.8℃ 67次/分 16次/分 120/74mmHg 158.2cm 54.8kg 21.9
  - HEENT :
    - Eyes: no anemic conjunctiva, no icteric sclera, normal EOM
    - ENT: no otorrhea, no rhinorrhea, no erythematous mucosa, no injected tonsil, no deformity
  - Neck: supple, freely movable, trachea midline,no deformity, no engorged jugular vein , no palpable thyroid or lymph node enlargement
  - Chest:
    - Inspection: Grossly normal, symmetric and fully expansion, no accessory muscle used, no operation scar, no spider angiomata, no gynecomastia
    - Palpation: no palpable mass or nodule, no subcutaneous emphysema, no tenderness
    - Ausculation: clear breathing sound
  - Heart:
    - Percussion: normal heart size
    - Ausculation: regular heart beat, normal S1, S2, no gallop, no murmur
  - Abdomen:
    - Inspection: in
      - flat contour, symetrically, no scar, no stria, no dilated veins, no rashes
    - Ausculation: normoactive bowel sound, no abdominal bruits
    - palpation: soft, no muscle guarding or rigidity, no local tenderness, no rebounding pain, no palpable mass, no palpable pulsation

- ◦ Percussion: no shifting dulllness, no hepatomegaly, no splenomagaly
-

Peripheral pulsation :	CA	BA	RA	FA	PA	PTA	DPA
Right side :	++	++	++	++	++	++	++/+++
Left side :	++	++	++	++	++	++	++/+++

- Genito-anus: no tenderness or palpable mass lesion or rectal shelf, normal external genitalia, normal anal tone, brown stool

CRS CONDITION

-
    - ◦ Lung

手術日期及方法

- 手術日期及方法20210610 Robot Assisted Low Anterior Resection

住院治療經過

- After admisison, pre op survey was OK and operation was done smoothly. She toelrated the procedure well and could stand oral feeding gradually. Under relatively stable conditions, she will be discharged.

合併症

一般檢查

- 累積報告 :

DATE	TIME	ALB	TP	BIL.T	BIL.D	ALKP	AST	ALT	LDH	AMY	LIPASE	NH3	Ferritin
1100610	2011			1.55				13					

DATE	TIME	WBC	RBC	HGB	HCT	MCV	PLT	BLST	NEUT	BAND	SEG	LYM	MON	EOS	BAS
1100610	2012	7420		10.8	32.1		171		92.4			5.1	2.4	0.0	0.1

DATE	TIME	NA	K	CL	BUN	CREAT	eGFR	CA	Mg	P	CRP	ESR	PCT	D-Dimer	LACTATE
1100610	2011	139	3.5	107	12	0.65	102.55	8.1							

特殊檢查

病理報告

其它

出院時情況

- 3.改門診治療

出院指示

-

藥名	劑量	劑量單位	用法	頻次	數量	數量單位	現況	天數	單位
(□)Takepron OD tab 30mg	1	TAB	PO	QDAC	14	TAB	TH	14	天
Ultracet tab	1	TAB	PO	QID	20	TAB	TH	5	天
Mag. Oxide tab	2	TAB	PO	TID	84	TAB	TH	14	天
$Lederscon tab	1	TAB	PO	QIDPRN	56	TAB	TH	14	天

出院計劃

主治醫師 2062F 蔣鋒帆 / 住院醫師 2309E 古朋育

正式摘要(電子簽章：**000000218842040**)

W95 / W95

doctorRank:B
